# Supplementary material for: Physics‐Aware Machine‐Learning‐Driven Inverse Design of Broadband Ultra‐Open Acoustic Metamaterials
Source: Adv Sci (Weinh). 2026 Aug 12:e76894. Online ahead of print. doi: 10.1002/advs.76894 (PMC13464556; doi:10.1002/advs.76894)
Supplement: Supplementary file 1 [file ADVS-9999-e76894-s001.docx]

Supplementary Information

**Physics-Aware Machine-Learning-Driven Inverse Design of Broadband Ultra-Open Acoustic Metamaterials**

Zhiwei Yang^1,2†^, Mengyu Li^1,2†^, Xiaohang Xie^1,2^, Ao Chen^1,2^, Thomas G. Bifano^1,2,3,4,5^, and Xin Zhang^1,2,3,4,5*^

^1^Department of Mechanical Engineering, Boston University, Boston, MA 02215, United States.

^2^Photonics Center, Boston University, Boston, MA 02215, United States.

^3^Division of Materials Science & Engineering, Boston University, Boston, MA 02215, United States.

^4^Department of Electrical & Computer Engineering, Boston University, Boston, MA 02215, United States.

^5^Department of Biomedical Engineering, Boston University, Boston, MA 02215, United States.

*Corresponding authors. E-mail(s): xinz@bu.edu

^†^These authors contributed equally to this work.

**Supplementary Note 1:** **Analytical transmittance of bilayer scattering system calculated based on Green’s function**

Green’s function $G(r, x; r_{0}, x_{0})$ within a semi-infinite circular duct terminating at $x = 0$ can be derived as the solution of the Helmholtz equation,

$$\nabla^{2}G(r, x;r_{0}, x_{0})+k_{0}^{2}G(r, x;r_{0}, x_{0})=-\frac{\delta\left( r-r_{0} \right)\delta\left( x-x_{0} \right)}{2\pi r}$$

where $\delta$ represents the Dirac function. The Green’s function with respect to the incident and transmitted side of the bilayer scattering system can be obtained as:

$$x\leq0:G_{1}(r, x;r_{0}, x_{0})=\sum_{n=0}^{n=\infty} \frac{\varphi_{n}\left( r_{0} \right)\varphi_{n}\left( r \right)}{-2i\pi R^{2}\sqrt{k_{0}^{2}-k_{n}^{2}}}(e^{i\sqrt{k_{0}^{2}-k_{n}^{2}}\left| x-x_{0} \right|}+e^{i\sqrt{k_{0}^{2}-k_{n}^{2}}\left| x-x_{0} \right|})$$

$$x\geq t :G_{2}(r, x;r_{0}, x_{0}) =\sum_{n=0}^{n=\infty} \frac{\varphi_{n}\left( r_{0} \right)\varphi_{n}\left( r \right)}{-2i\pi R^{2}\sqrt{k_{0}^{2}-k_{n}^{2}}}(e^{i\sqrt{k_{0}^{2}-k_{n}^{2}}\left| x-x_{0} \right|}+e^{i\sqrt{k_{0}^{2}-k_{n}^{2}}\left| x-x_{0}-2t \right|})$$

where $\varphi_{n}\left( r \right)=J_{0}(k_{n}r)/J_{0}(k_{n}R)$ and the wavenumbers $k_{n}$ are determined by the root condition $J_{1}(k_{n}R)=0$. Here, in which $J_{0}(\cdot)$ and $J_{1}(\cdot)$ are Bessel functions. $R$ and $t$ are the duct radius and metamaterial’s thickness, respectively.

Utilizing the Kirchhoff-Helmholtz integral, the pressure field on the left side of the scattering system ($x=0$) may be written as:

$$p\left( r, 0 \right)=2+2\pi\int_{r_{11}}^{r_{12}} G_{1}\left( r, x;r_{0}, 0 \right)\left. \frac{\partial p\left( r_{0}, x_{0} \right)}{\partial x_{0}} \right|_{x_{0}=0}r_{0}dr_{0}+2\pi\int_{r_{21}}^{r_{22}} G_{1}\left( r, x;r_{0}, 0 \right)\left. \frac{\partial p\left( r_{0}, x_{0} \right)}{\partial x_{0}} \right|_{x_{0}=0}r_{0}dr_{0}$$

By applying the conservation of the momentum,

$$i\omega\rho_{0}u=-\partial p/\partial x$$

The equation becomes

$$p\left( r, 0 \right)=2+2\pi i\omega\rho_{0}\int_{r_{11}}^{r_{12}} G_{1}\left( r, x;r_{0}, 0 \right)u_{10}r_{0}dr_{0}+2\pi i\omega\rho_{0}\int_{r_{21}}^{r_{22}} G_{1}\left( r, x;r_{0}, 0 \right)u_{20}r_{0}dr_{0}$$

The average pressure field can be expressed by the average velocity

$$P_{10}=2+\frac{4i\rho_{0}\omega U_{10}}{S_{1}^{2}}\int_{r_{11}}^{r_{12}} \int_{r_{11}}^{r_{12}} G_{1}\left( r, x;r_{0}, 0 \right)r_{0}dr_{0}rdr+\frac{4i\rho_{0}\omega U_{20}}{S_{1}S_{2}}\int_{r_{11}}^{r_{12}} \int_{r_{21}}^{r_{22}} G_{1}\left( r, x;r_{0}, 0 \right)r_{0}dr_{0}rdr$$

$$P_{20}=2+\frac{4i\rho_{0}\omega U_{10}}{S_{1}S_{2}}\int_{r_{21}}^{r_{22}} \int_{r_{11}}^{r_{12}} G_{1}\left( r, x;r_{0}, 0 \right)r_{0}dr_{0}rdr+\frac{4i\rho_{0}\omega U_{20}}{S_{2}^{2}}\int_{r_{21}}^{r_{22}} \int_{r_{21}}^{r_{22}} G_{1}\left( r, x;r_{0}, 0 \right)r_{0}dr_{0}rdr$$

and $S_{i}$ denotes the cross-sectional area of region $i$. Similarly, when $x=t$, following the same procedure and employing the Green’s function $G_{2}(r, x;r_{0}, x_{0})$, the pressure may be derived as

$$P_{1t}=-\frac{4i\rho_{0}\omega U_{1t}}{S_{1}^{2}}\int_{r_{11}}^{r_{12}} \int_{r_{11}}^{r_{12}} G_{2}\left( r, x;r_{0}, t \right)r_{0}dr_{0}rdr-\frac{4i\rho_{0}\omega U_{2t}}{S_{1}S_{2}}\int_{r_{11}}^{r_{12}} \int_{r_{21}}^{r_{22}} G_{2}\left( r, x;r_{0}, t \right)r_{0}dr_{0}rdr$$

$$P_{2t}=-\frac{4i\rho_{0}\omega U_{1t}}{S_{1}S_{2}}\int_{r_{21}}^{r_{22}} \int_{r_{11}}^{r_{12}} G_{2}\left( r, x;r_{0}, t \right)r_{0}dr_{0}rdr-\frac{4i\rho_{0}\omega U_{2t}}{S_{2}^{2}}\int_{r_{21}}^{r_{22}} \int_{r_{21}}^{r_{22}} G_{2}\left( r, x;r_{0}, t \right)r_{0}dr_{0}rdr$$

Next, considering that regions 1 and 2 are separated by a hard boundary, the transfer matrices can be derived in a decoupled fashion, which provides a relationship between the inlet and outlet pressures and velocity fields. Assuming that region 1 and region 2 are narrow, the inner averaged pressure and volume velocity fields can be, respectively, estimated by the fundamental mode as:

$$P_{m}(x)=A_{1m}e^{ik_{0}n_{m}x}+A_{2m}e^{-ik_{0}n_{m}x}$$

$$U_{m}(x)=A_{1m}e^{ik_{0}n_{m}x}/Z_{m}-A_{2m}e^{-ik_{0}n_{m}x}/Z_{m}$$

where $A_{1m}$ and $A_{2m}$ are constant coefficients associated with the incoming and outgoing waves, respectively. $n_{m}$ and $Z_{m}$ (m=1,2) are the effective refractive indexes and effective acoustic impedances of region m, respectively. Subsequently, one may find the transfer matrices for region 1 and region 2 as

$$\left[ \begin{aligned} P_{m0} \\ u_{m0} \end{aligned} \right]=\left[ \begin{matrix} cos(k_{0}n_{m}t) & -iZ_{m}sin(k_{0}n_{m}t) \\ -isin(k_{0}n_{m}t)/Z_{m} & cos(k_{0}n_{m}t) \end{matrix} \right]\left[ \begin{aligned} P_{mt} \\ u_{mt} \end{aligned} \right]$$

where $u_{1t}=U_{1t}/S_{1}$ and $u_{2t}=U_{2t}/S_{2}$.

Combining these equations provide a solvable system of eight equations from which averaged pressure and velocity terms defined as eight variables may readily be calculated. The outlet volume velocities $U_{1t}$ and $U_{2t}$ are obtained by solving the following linear system:

$$M\left[ \begin{aligned} P_{10} \\ P_{20} \\ P_{1t} \\ P_{2t} \\ U_{10} \\ U_{20} \\ U_{1t} \\ U_{2t} \end{aligned} \right]=\left[ \begin{aligned} 2 \\ 2 \\ 0 \\ 0 \\ 0 \\ 0 \\ 0 \\ 0 \end{aligned} \right]$$

Here, $M$ is an $8\times8$coefficient matrix given by

$$M=\left[ \begin{matrix} 1 & 0 & 0 & 0 & -g_{11} & -g_{12} & 0 & 0 \\ 0 & 1 & 0 & 0 & -g_{21} & -g_{22} & 0 & 0 \\ 0 & 0 & 1 & 0 & 0 & 0 & g_{11} & g_{12} \\ 0 & 0 & 0 & 1 & 0 & 0 & g_{21} & g_{22} \\ 1 & 0 & -cos(k_{0}n_{1}t) & 0 & 0 & 0 & iZ_{1}sin(k_{0}n_{1}t)/S_{1} & 0 \\ 0 & 0 & \frac{iS_{1}sin\left( k_{0}n_{1}t \right)}{Z_{1}} & 0 & 1 & 0 & -cos(k_{0}n_{1}t) & 0 \\ 0 & 1 & 0 & -cos(k_{0}n_{2}t) & 0 & 0 & 0 & \frac{iZ_{2}sin\left( k_{0}n_{2}t \right)}{S_{2}} \\ 0 & 0 & 0 & \frac{isin\left( k_{0}n_{2}t \right)S_{2}}{Z_{2}} & 0 & 1 & 0 & -cos(k_{0}n_{2}t) \end{matrix} \right]$$

The coupling terms $g_{ij}$ are computed from the Green’s function integral,

$$g_{ij}=\frac{4\pi\rho_{1}k_{0}c_{1}}{S_{i}S_{j}}\int_{r=r_{i1}}^{r=r_{i2}} \int_{r_{0}=r_{j1}}^{r_{0}=r_{j2}} \sum_{n=0}^{\infty} \frac{\varphi_{n}(r_{0})\varphi_{n}(r)}{-R^{2}\sqrt{k_{0}^{2}-k_{n}^{2}}}rr_{0}drdr_{0}$$

The normalized volume velocities, $\bar{u}_{10}$, $\bar{u}_{1t}$, $\bar{u}_{20}$, $\bar{u}_{2t}$, are defined by $\bar{u}=(\rho_{0}c_{0}/\pi R^{2})U$. The transmission and reflection pressures in the far field can be derived as:

$$p_{t}(x)=\left( \bar{u}_{1t}+\bar{u}_{2t} \right)e^{ik_{0}\left( x-t \right)}$$

$$p_{r}\left( x \right)=\left( 1-\bar{u}_{10}-\bar{u}_{20} \right)e^{-ik_{0}x}$$

As a result, the power-transmission and -reflection coefficients are readily obtained as

$$T=\left| \bar{u}_{1t}+\bar{u}_{2t} \right|^{2}$$

$$R=\left| 1-\bar{u}_{10}-\bar{u}_{20} \right|^{2}$$

**Supplementary Note 2**: **Detailed introduction of the two stage forward predictions framework**

In Stage 1, the DenseConv Upsampler transforms the six-parameter input into a high-resolution 1D latent representation by predicting the calculated frequency domain transmission spectra as training targets. Specifically, the input vector of dimension 6 and batch size $B$ ($B=256$) is first linearly projected and patched (an inverse convolutional network) through a fully connected network:

$$\begin{aligned} R^{6}\to R^{C\cdot L_{0}} \end{aligned}$$

and reshaped into an initial feature map of size, $(C, L_{0})$. The latent sequence is then progressively expanded through four transposed convolution upsampling stages, increasing the embedding length from $L_{0}=16$ to $L_{4}=256$. Each upsampling stage employs the same building block and consists of a ConvTranspose1D layer with an upsampling factor of 2, followed by a DenseBlock1D containing three densely connected convolutional layers, and a Squeeze-and-Excitation (SE) attention module that performs channel-wise reweighting:

$$\begin{aligned} w_{c}=\sigma(FC_{2}(ReLU(FC_{1}(GAP(x_{c}))))) \end{aligned}$$

where $GAP(x_{c})$ denotes global average pooling applied to channel $c$, $ReLU(\cdot)$ is the rectified linear unit, $FC_{1}$ and $FC_{2}$ denote the first and second fully connected layer, and $\sigma(\cdot)$ is the sigmoid activation. This attention mechanism adaptively emphasizes informative channels during feature expansion. The DenseConv Upsampler outputs a latent embedding of size (64, 256), which is further processed by 1x1 convolutional blocks (conv1×1 blocks), followed by flattening and linear projection to generate the target transmission spectrum during Stage 1 training. Alternatively, this latent embedding can be emitted as a latent representation for subsequent refinement in Stage 2. Owing to its progressive transposed-convolution upsampling architecture, Stage 1 is particularly well suited for coarse spectral prediction, effectively mapping a low-dimensional design vector to a high-resolution spectral representation while capturing the global, low-frequency characteristics of the response.

In Stage 2, the U-Net Upsampler refines the latent embedding to further improve high-frequency accuracy and reconstruct sharp spectral transitions. The U-Net Upsampler takes the Stage 1 latent output of dimension (64, 256) as input and processes it using a full 1D U-Net encoder–decoder architecture with skip. Similar to the original 2D U-Net, the encoder-decoder structure comprises three hierarchical levels. Downsampling is performed using 1D convolution block followed by 1D max pooling, while upsampling is implemented using 1D transpose convolution blocks with skip-connection concatenation. After refinement, the latent sequence retains the same dimensionality (64, 256) and is first flattened, then processed by a multi-layer perceptron (MLP), and finally linearly projected to produce the final predicted transmission spectrum. By preserving the full latent resolution throughout the refinement process, Stage 2 is particularly well suited for spectral refinement, enabling accurate correction of fine-scale features while maintaining the expressive capacity of the latent representation.

The forward prediction network is trained using a two-stage curriculum strategy designed to stabilize optimization and improve sharp feature predictions. In Stage 1, during the first $N_{1}$ epochs, the DenseConv Upsampler is fully trained, while the U-Net Upsampler is frozen. In this stage, the DenseConv Upsampler acts as a trainable encoder that learns a coarse but physically consistent mapping from the design parameters to the frequency-domain response,

$$x \to\hat{y}_{Dense}$$

with the loss applied directly to the predicted spectrum. After Stage 1 convergence, the DenseConv Upsampler is frozen and Stage 2 training is initiated. In Stage 2, the U-Net Upsampler together with the MLP head is trained on latent features $z$ extracted from the final Dense Convolution Block.

$$\begin{aligned} z=extract\_latent(x) \end{aligned}$$

to refine the mapping

$$x \to\hat{y}_{unet\_refined}$$

For both stages, the mean square error (MSE) loss function is employed, and optimization is performed using the AdamW optimizer. In this work, both Stage 1 and Stage 2 are trained for $N_{1}=N_{2}=1000$ epochs.

**Supplementary Note 3: Ablation study of the two-stage versus end-to-end training strategy**

To verify that the proposed training strategy provides benefits beyond the network architecture itself, an ablation study was performed by comparing the proposed two-stage optimization with direct end-to-end training. In both cases, the identical DenseConv–U-Net architecture was employed. The only difference was the optimization strategy. For the end-to-end model, the DenseConv Upsampler, U-Net Upsampler, and prediction head were optimized simultaneously from random initialization using a single MSE loss. For the proposed approach, the DenseConv Upsampler was first trained independently for coarse spectral prediction, after which its weights were frozen and the U-Net Upsampler was trained to refine the latent representation.

Both models were trained using the same dataset consisting of 200,000 analytically generated samples with an identical 80/20 train–test split. The training and testing datasets were controlled using identical sample indices to ensure a fair comparison. All optimization settings, including learning rate schedule, optimizer, batch size, and total number of training epochs 2,000, were kept identical between the two methods.

Figure S1 compares the training and testing losses of the two optimization strategies. The proposed two-stage strategy consistently converges to lower training and testing losses than direct end-to-end optimization while exhibiting substantially smoother convergence throughout training. In addition, the total training time required for the proposed strategy was approximately 11 h, compared with approximately 35 h for end-to-end training under identical hardware conditions. These results indicate that freezing the first-stage DenseConv Upsampler preserves the coarse global spectral representation while allowing the U-Net to focus exclusively on high-resolution spectral refinement, leading to improved optimization stability, prediction accuracy, and computational efficiency.

**Figure S1.** **Comparison between the proposed two-stage training strategy and direct end-to-end training.** (a) Training loss comparison. (b) Test loss comparison. Both methods used the same network architecture, 200,000-sample dataset, identical 80/20 train–test split, and matched optimization settings. The two-stage strategy achieves lower final training and test losses with reduced fluctuations, indicating improved prediction accuracy, optimization stability, and generalization.

**Supplementary Note 4: Dataset generation**

To construct the transmission-response dataset, UAS configurations are generated by random sampling in the six-dimensional design space.

$$x=\{r_{1, 1}, r_{1, 2}, r_{2, 1}, r_{2,2}, t, n_{2}\}\in\mathbb{R}^{6}$$

The radial parameters are sampled using a transformed-variable formulation to enforce the geometric ordering

$$0\leq r_{1,1}<r_{1,2}<r_{2,1}<r_{2,2}\leq R$$

Let $d_{1}$, $d_{2}$, and $d_{3}$ denote the positive radial increments defined by

$$d_{1}=r_{1,2}-r_{1,1}$$

$$d_{2}=r_{2,1}-r_{1,2}$$

$$d_{3}=r_{2,2}-r_{2,1}$$

These increments are independently sampled from a uniform distribution,

$$d_{j}\mathcal{\sim U}\left( d_{\min},d_{\max} \right), j=1, 2, 3$$

where $\mathcal{U(}a,b)$ denotes a continuous uniform distribution over the interval $[a, b]$, and $d_{\min}$ and $d_{\max}$ represent the minimum and maximum allowable gaps, respectively.

The innermost radius is sampled as

$$r_{1,1}\mathcal{\sim U}(0, R-3d_{\min}),$$

and the remaining radii are constructed sequentially:

$$r_{1,2}=r_{1,1}+d_{1}$$

$$r_{2,1}=r_{1,2}+d_{2}$$

$$r_{2,2}=r_{2,1}+d_{3}$$

Only samples satisfying the constraint $r_{2,2}<R$ are retained.

The spectral parameters are sampled independently as

$$t\sim\mathcal{U}\left( t_{\min}, t_{\max} \right),$$

$$\theta\sim\mathcal{U(}\theta_{\min}, \theta_{\max}),$$

where $t_{\min}$ and $t_{\max}$ define the thickness range, and $\theta_{\min}$ and $\theta_{\max}$ define the effective tilting-angle range, the effective refractive index is then computed from the tilting angle using

$$n_{2}=\frac{1}{\sin\theta}$$

Each sampled configuration can therefore be written as

$$x=\{r_{1, 1}, r_{1, 1}+d_{1}, r_{1, 1}+d_{1}+d_{2}, r_{2,2}+d_{1}+d_{2}+d_{3}, t, \frac{1}{\sin\theta}\}$$

To avoid duplicate configurations arising from floating-point precision, each sampled vector is discretized using a prescribed precision $\varepsilon$,

$$\tilde{x}=\mathrm{round}(\frac{x}{\varepsilon})\varepsilon,$$

where $\mathrm{round}(\cdot)$ denotes element-wise rounding to the nearest integer, and where $\varepsilon={10}^{-8}$ denotes the discretization precision. Only unique samples are retained in the dataset and total 200,000 samples are generated for simulation.

**Supplementary Note 5: Diversity-aware selection and final hard-threshold filtering**

To further improve the robustness and practical relevance of the inverse prediction results, two additional mechanisms are incorporated after the stage-wise optimization procedure: diversity-aware candidate selection and final hard-threshold filtering.

First, instead of selecting candidates solely based on the objective value, a diversity-aware selection strategy is employed at the end of each stage. Given a candidate set $X_{s}$ and the corresponding per-sample objective values $\mathcal{J(}x)$, candidates are first ranked in ascending order of loss. A greedy selection procedure is then applied to construct a subset of size $K_{s}$ such that geometric redundancy is reduced. Specifically, for each candidate $x^{(i)}$, similarity to previously selected candidates is evaluated over the radial parameter subset

$$(r_{1,1}, r_{1,2}, r_{2,1}, r_{2,2}),$$

and a candidate is accepted only if it satisfies

$$\left| x_{d}^{\left( i \right)}-x_{d}^{\left( j \right)} \right|>\delta_{d}, d\in\{1,2,3,4\},$$

for all previously selected candidates $x^{(j)}$, where $\delta_{d}$ denotes a predefined tolerance for each dimension. In this work, the tolerance values are set uniformly as $\delta_{d}=0.001$ for $d\in\{1,2,3,4\}$. This procedure ensures that the retained candidates are both low-loss and geometrically diverse, preventing collapse of the population to a narrow region of the design space.

After completion of all optimization stages, a final hard-threshold filtering step is applied to enforce strict band-limited performance in the original frequency domain. Let $f\in[f_{\mathrm{low}}^{\mathrm{hard}}, f_{\mathrm{high}}^{\mathrm{hard}}]$ denote a prescribed frequency band. For each candidate $x$, the predicted transmission spectrum $\hat{T}(f;x)$ is evaluated, and the maximum transmission within the band is computed as

$$T_{\max}\left( x \right)=\max_{f\in[f_{\mathrm{low}}^{\mathrm{hard}}, f_{\mathrm{high}}^{\mathrm{hard}}]} \hat{T}(f;x)$$

Only candidates satisfying the strict constraint

$$T_{\max}(x)\leq\tau_{\mathrm{hard}}$$

are retained, where $\tau_{\mathrm{hard}}$ is a prescribed transmission threshold. Note $\tau_{\mathrm{hard}}$ may differ from the optimization threshold $\tau$ used in the objective function. This filtering step is applied only once after the final stage and does not affect the optimization trajectory.

The combination of diversity-aware selection and post hoc hard-threshold filtering enables the framework to identify multiple geometrically distinct solutions while ensuring that the final candidate set strictly satisfies the desired band-limited attenuation criteria.

**Supplementary Note 6: Comparison for the machine learning (ML) inverse design and the** **Finite Element Method (FEM) approach**

Here, we compare the computational cost of the machine-learning (ML) approach with that of the conventional Finite Element Method (FEM) parameter-sweeping approach. Once the dataset has been generated, training the neural network requires only several hours (approximately 11h on an NVIDIA RTX 3090 Ti GPU), and inverse design for a given target spectrum can be completed within minutes. By eliminating the need for iterative FEM simulations during the design stage, the proposed framework substantially reduces the computational time required for design construction. Moreover, once trained, the modal can be reused for different target responses and can generate candidate acoustic metamaterial (AMM) designs within minutes, in contrast to the time-consuming FEM-based optimization procedures.

Table S1 summarizes the computational budget for both approaches. For the ML framework, the total time consists of three main stages: dataset generation, network training, and design validation. The dataset is constructed using the effective medium theory (EMT) model rather than full-wave simulations, enabling efficient generation of a large dataset. Dataset construction dominates the total computational cost, accounting for approximately 98% of the total ML runtime, and involves the calculation of 200,000 samples. In comparison, network training requires roughly half a day. During the inverse-design stage, 100 candidate structures are generated for each target spectrum. To convert the predicted effective refractive index of the Region 2 into a realizable geometry (coil channel), the tilting angle must be determined, which requires approximately 20 minutes per design for validation simulations.

For the conventional FEM approach, a parameter-sweeping strategy was adopted. Each parameter was discretized into 20 values (10 values with coarse mesh and 10 value values with fine mesh). Within the considered design space (six parameters), an exhaustive FEM traversal would require approximately ${10}^{5}$ days. Even with one thousand parallel processors, the estimated computational time would still be in the order of ${10}^{2}$ days, highlighting the impracticality of brute-force parameter searches. In practice, parameter variations are often interdependent, which can further increase the required computational time.

| **Duration of machine learning inverse design** | | |
| --- | --- | --- |
|  | **Time (s)** | **Time (d)** |
| **Dataset construction** | $\boldsymbol{2.40\times1}\boldsymbol{0}^{\boldsymbol{6}}$ | $\boldsymbol{2.78\times10}$ |
| **Training** | $\boldsymbol{3.96\times1}\boldsymbol{0}^{\boldsymbol{4}}$ | $\boldsymbol{4.58\times1}\boldsymbol{0}^{\boldsymbol{-1}}$ |
| **Generation (100 candidates)** | $\boldsymbol{3.27\times1}\boldsymbol{0}^{\boldsymbol{2}}$ | $\boldsymbol{3.78\times1}\boldsymbol{0}^{\boldsymbol{-3}}$ |
| **Validation through FEM** | $\boldsymbol{1.20\times1}\boldsymbol{0}^{\boldsymbol{3}}$ | $\boldsymbol{1.39\times1}\boldsymbol{0}^{\boldsymbol{-2}}$ |
| **Total** | $\boldsymbol{2.44\times1}\boldsymbol{0}^{\boldsymbol{6}}$ | $\boldsymbol{2.83\times10}$ |
| **Duration of FEM approach** | | |
|  | **Time (s)** | **Time (d)** |
| **3 parameters** | $\boldsymbol{9.60\times1}\boldsymbol{0}^{\boldsymbol{6}}$ | $\boldsymbol{1.11\times1}\boldsymbol{0}^{\boldsymbol{2}}$ |
| **4 parameters** | $\boldsymbol{1.92\times1}\boldsymbol{0}^{\boldsymbol{8}}$ | $\boldsymbol{2.22\times1}\boldsymbol{0}^{\boldsymbol{3}}$ |
| **5 parameters** | $\boldsymbol{3.84\times1}\boldsymbol{0}^{\boldsymbol{9}}$ | $\boldsymbol{4.44\times1}\boldsymbol{0}^{\boldsymbol{4}}$ |
| **6 parameters** | $\boldsymbol{7.68\times1}\boldsymbol{0}^{\boldsymbol{10}}$ | $\boldsymbol{8.89\times1}\boldsymbol{0}^{\boldsymbol{5}}$ |

Table S1. Breakdown of the computational budget for the machine learning approach, including generation, training, validation of designs per spectrum, compared to the running time of multi-parameter simulations using FEM, on an AMD Ryzen 9 5900X 12-Core Processor CPU with 64 GB of RAM and an NVIDIA RTX 3090 Ti GPU.

**Supplementary Note 7: Quantitative analysis of the coupling between spectral and radial parameters**

In our design framework, $n_{2}$ and $t$ are treated as spectral parameters, while $k_{1,1}$, $k_{1,2}$, and $k_{2,2}$ are treated as radial parameters. Therefore, the relationship between $t$ and $k_{1,1}$ was used as a representative metric for spectral–radial coupling.

Specifically, $t$ was varied from 0.03 to 0.07 m, and $k_{1,1}$ was varied from 0 to 0.4, while the other parameters were fixed as $k_{1,2}=0.3$, $k_{2,2}=0.3$, and $n_{2}=3.5$. For each parameter combination, the transmission spectrum was calculated. We then selected the parameter sets satisfying $T_{f=1000 Hz}<0.01$, corresponding to the target attenuation condition at the representative frequency of $1000 Hz$, for further analysis.

As shown in Fig. S2(a), a linear regression was performed between $t$ and $k_{1,1}$ among the selected parameter sets. The fitted relationship was $k_{1,1}=-193.5t+10.3,$ with $R^{2}=0.264$. This low $R^{2}$ value indicates that only 26.4% of the variation in $k_{1,1}$ can be explained by the variation in $t$ under the selected transmission criterion. This result provides quantitative evidence of weak linear correlation between the representative spectral parameter $t$ and the radial parameter $k_{1,1}$, supporting weak coupling between the spectral and radial parameter groups.

For comparison, we also examined the coupling within the spectral-parameter group by sweeping $t$ and $n_{2}$, as shown in Fig. S2 (b). In this analysis, $t$ was varied from 0.03 to 0.07 m, and $n_{2}$ was varied from 2.5 to 5.0, while the radial parameters were fixed as $k_{1,1}=0.1$, $k_{1,2}=0.3$, and $k_{2,2}=0.3$. After applying the same selection criterion of $T_{f=1000 Hz}<0.01$, the fitted relationship between $t$ and $1/n_{2}$ was: $1/n_{2}=5.040t+0.022$, with $R^{2}$=0.998. This high $R^{2}$ value confirms the expected strong correlation between the two spectral parameters. In contrast, the much lower $R^{2}$ value for the $t$–$k_{1,1}$ relationship indicates weak spectral–radial coupling. This comparison provides quantitative support for the proposed functional decoupling between the spectral and radial parameter groups.

Figure S2. Quantitative analysis of the coupling between spectral and radial parameters. (a) Relationship between the spectral parameter $t$ and the radial parameter $k_{1,1}$ among the parameter sets satisfying $T_{f=1000 Hz}<0.01$. The low $R^{2}$ value indicates weak spectral–radial correlation. (b) Relationship between the spectral parameters $t$ and $n_{2}$ among the selected parameter sets. The high $R^{2}$ value indicates strong correlation within the spectral-parameter group.

**Supplementary Note 8: Validation of the Analytical Green’s Function Model Using Full-Wave FEM Simulations**

To exclude potential systematic discrepancies between the analytical model and the full-wave FEM simulations, we performed an additional statistical validation. Specifically, 100 designs were randomly sampled from the predefined design space, and their transmission spectra were computed using both the analytical Green’s function model and full-wave FEM simulations. The selected samples were distributed throughout the investigated design space. Figure S3 shows the distribution of the 100 selected designs within the design space, while Fig. S4–S8 compare the corresponding transmission spectra obtained using the analytical model and FEM.

To quantitatively evaluate the agreement between the two methods, we calculated the mean squared error (MSE) between the transmission coefficients obtained by the analytical model and FEM over all sampled frequency points. For each design, the MSE was calculated as

$$MSE=\frac{1}{N}\sum_{i=1}^{N} \left( T_{FEM,i}-T_{Analytical, i} \right)^{2}$$

where $N$ is the number of frequency points, and $T_{FEM,i}$ and $T_{Analytical, i}$ represent the FEM and analytical results at the $i$th frequency point., respectively.

The distribution of the MSE values for the 100 individual designs is presented in Fig. S9. The mean MSE was $1.26\times{10}^{-3}$, and the maximum MSE was 0.01. These results indicate close agreement between the analytical model and FEM across the sampled design space, rather than only for a few selectively chosen designs. They therefore support the reliability of the analytical Green’s function model for generating the 200,000-sample dataset within the investigated parameter range.

Nevertheless, we acknowledge that the analytical Green’s function model remains an approximation of the full-wave system and is applicable under the assumptions described in the Supplementary Note 1.

Figure S3. Distribution of the 100 randomly sampled designs within the predefined design space.

Figure S4. Comparison of the transmission spectra obtained using the analytical Green’s function model and full-wave FEM for randomly sampled designs No. 1–20.

Figure S5. Comparison of the transmission spectra obtained using the analytical Green’s function model and full-wave FEM for randomly sampled designs No. 21–40.

Figure S6. Comparison of the transmission spectra obtained using the analytical Green’s function model and full-wave FEM for randomly sampled designs No. 41–60.

Figure S7. Comparison of the transmission spectra obtained using the analytical Green’s function model and full-wave FEM for randomly sampled designs No. 61–80.

Figure S8. Comparison of the transmission spectra obtained using the analytical Green’s function model and full-wave FEM for randomly sampled designs No. 81–100.

Figure S9. Mean squared error between the transmission coefficients obtained using the analytical Green’s function model and full-wave FEM for the 100 randomly sampled designs.

**Supplementary Note 9: Coefficient uncertainties and the coefficients of determination for all fitted lines.**

To determine whether the identified lines in Figure 5 are genuinely distinct rather than artifacts of the clustering procedure, here, we report the coefficient uncertainties and the coefficients of determination $R^{2}$ for all fitted lines, as summarized in Table S2.

The $R^{2}$ values range from 0.986 to 0.995, indicating that the linear models provide close fits to the corresponding datasets. In addition, the standard errors of the fitted slopes and intercepts provide a quantitative basis for evaluating the separation between the UAS-1 and UAS-2 fitting lines at each value of $\tau$. The differences in the fitted slopes and intercepts are substantially larger than their corresponding standard errors, indicating that the two linear relationships are distinguishable within the investigated parameter range. Together with the consistently high $R^{2}$ values, these results suggest that the identified fitting lines reflect robust relationships in the data rather than artifacts introduced by the clustering procedure.

|  | Threshold | Slope | Intercept | $R^{2}$ |
| --- | --- | --- | --- | --- |
| UAS-1 | $\tau=$ 0.1 | 2.881±0.053 | 0.382±0.004 | 0.993 |
| UAS-2 | $\tau=$ 0.1 | 3.843±0.038 | -0.634±0.010 | 0.994 |
| UAS-1 | $\tau=$ 0.05 | 2.731±0.033 | 0.257±0.003 | 0.992 |
| UAS-2 | $\tau=$ 0.05 | 3.506±0.068 | -0.372±0.013 | 0.986 |
| UAS-1 | $\tau=$ 0.03 | 2.638±0.027 | 0.199±0.003 | 0.993 |
| UAS-2 | $\tau=$ 0.03 | 3.513±0.044 | -0.271±0.008 | 0.995 |

Table S2. Slopes, intercepts, and coefficients of determination $R^{2}$ for the linear fits of $k_{2,2}$ as a function of $k_{1,2}$ under UAS-1 and UAS-2 at $\tau=$ 0.1, 0.05 and 0.03. Values are reported as fitted coefficient with standard error.

**Supplementary Note 10: Pressure drop and airflow-dependent transmission-loss spectra**

The wind velocity ratio ($v_{w}/v_{wo}$) alone does not fully characterize the ventilation performance of a ventilated acoustic silencer, because it does not directly reflect the pressure drop introduced by the structure. In this work, the wind velocity ratio is used as an intuitive airflow-retention metric, while pressure drop is included as a complementary indicator of ventilation resistance. Airflow-dependent transmission-loss spectra are further provided to evaluate the acoustic performance under airflow conditions.

To address this concern, we performed additional COMSOL simulations under airflow conditions. In the same simulation framework, we extracted the inlet and outlet static pressures, calculated the pressure drop, and simulated the transmission loss under different inlet airflow velocities. The transmission-loss spectra were calculated for $Ma=$0, 0.01, 0.02, and 0.05, while the pressure drop was evaluated for the airflow cases of $Ma=$0.01, 0.02, and 0.05. The SST turbulence model and the linearized Navier–Stokes frequency-domain module were coupled to evaluate the airflow-dependent acoustic response.

The simulated pressure drop was calculated as $\Delta p=p_{\mathrm{in}}-p_{\mathrm{out}}$. It increased from 53.6 Pa at $Ma=0.01$, to 211.5 Pa at $Ma=0.02$, and 1346.3 Pa at $Ma=0.05$, which is consistent with the expected increase in flow resistance with inlet airflow velocity. These pressure-drop results provide an additional measure of ventilation resistance beyond the wind velocity ratio.

As shown in Fig. S10, increasing the inlet airflow velocity reduces the transmission-loss peaks and slightly shifts them toward lower frequencies, indicating that airflow can influence the acoustic response of the structure. Nevertheless, the overall broadband sound-attenuation profile remains largely maintained under the investigated airflow conditions, suggesting that the designed structure can still provide broadband acoustic attenuation in the presence of airflow.

Figure S10. Simulated transmission loss of the MLUOM at different inlet Mach numbers ($Ma=$0, 0.01, 0.02, and 0.05).
